# Draft Genome Sequence of *Corynebacterium striatum* 1961 BR-RJ/09, a Multidrug-Susceptible Strain Isolated from the Urine of a Hospitalized 37-Year-Old Female Patient

**DOI:** 10.1128/genomeA.00869-15

**Published:** 2015-08-06

**Authors:** Ana L. Mattos-Guaraldi, Luis C. Guimarães, Carolina S. Santos, Adonney A. O. Veras, Adriana R. Carneiro, Siomar C. Soares, Juliana N. Ramos, Cassius Souza, Veronica V. Vieira, Raphael Hirata, Vasco Azevedo, Luis G. C. Pacheco, Artur Silva, Rommel T. J. Ramos

**Affiliations:** a Faculty of Medical Sciences, Rio de Janeiro State University (UERJ), Rio de Janeiro, RJ, Brazil; b Institute of Biological Sciences, Federal University of Pará (UFPA), Belém, PA, Brazil; c Institute of Health Sciences, Federal University of Bahia (UFBA), Salvador, BA, Brazil; d Department of Immunology, Microbiology and Parasitology, Institute of Biological Sciences and Natural Sciences, Federal University of Triângulo Mineiro, Uberaba, MG, Brazil; e National Institute for Quality Control in Health, Oswaldo Cruz Foundation (INCQS–Fiocruz), Rio de Janeiro, RJ, Brazil; f Institute Oswaldo Cruz Foundation, Oswaldo Cruz Foundation (IOC–Fiocruz), Rio de Janeiro, RJ, Brazil; g Institute of Biological Sciences, Federal University of Minas Gerais (UFMG), Belo Horizonte, MG, Brazil

## Abstract

*Corynebacterium striatum* commonly colonizes the normal skin and nasopharyngeal tract of humans; however, this potentially pathogenic bacterium has been identified as the causative agent of several nosocomial infections. The current study describes the draft genome of strain 1961 BR-RJ/09, isolated from the urine of a hospitalized patient from Brazil.

## GENOME ANNOUNCEMENT

*Corynebacterium striatum* is a Gram-positive bacterium that belongs to the CMNR group, which includes species of the genera *Corynebacterium*, *Mycobacterium*, *Nocardia*, and *Rodococcus* (1). Pathogenic clones of *C. striatum*, mostly causing respiratory tract infections, have been identified in several nosocomial outbreaks reported in different countries (2–4). This microorganism has also been reported to be responsible for infections that include endocarditis, meningitis, and septic arthritis (2, 5). Many isolates of this emerging human pathogen already present a multidrug-resistant phenotype.

A recent study in Brazil (6) evaluated the antimicrobial susceptibility patterns of several *C. striatum* isolates recovered from hospitalized patients and, through pulsed-field gel electrophoresis (PFGE) analysis, identified clones associated with outbreaks and with multidrug resistance. In this context, the *C. striatum* 1961 BR-RJ/09 strain (PFGE type III, multidrug susceptible) was isolated from the urine of a hospitalized 37-year-old woman from Rio de Janeiro, Brazil (6). Genomic DNA of this isolate was extracted using the QIAamp DNA minikit (Qiagen) protocol, and genome sequencing was performed using an Ion Torrent Personal Genome Machine (PGM) System with a 318 chip and fragment libraries. The quality of reads was analyzed using the software FastQC (http://www.bioinformatics.babraham.ac.uk/projects/fastqc) and the *de novo* assembly was performed using MIRA v4.02 (7) and SPAdes v3.10 (8) assemblers, and curation to reduce the gaps was done with the Lasergene v11 Suite (DNAStar). The assembly produced 28 contigs with a total of 2,611,976 bp and 59.4% G+C content. The contigs were annotated using Rapid Annotations using Subsystems Technology (RAST) (9), which identified 2,455 protein-encoding genes and 68 RNA genes. Among the annotated protein-encoding genes, a *narKHJI* gene cluster was identified with high similarity to the *Corynebacterium diphtheriae* narKGHJI operon, which is responsible for nitrate reductase (10), suggesting a similar anaerobic growth mechanism. This genome is part of an ongoing study of the comparative genomics, pathogenicity, and vaccine and drug targets of the species.

### Nucleotide sequence accession number.

This genome has been deposited in GenBank under the accession number LAYR00000000.
